# A comparison of microsatellites and genome‐wide SNPs for the detection of admixture brings the first molecular evidence for hybridization between *Mustela eversmanii* and *M. putorius* (Mustelidae, Carnivora)

**DOI:** 10.1111/eva.13291

**Published:** 2021-08-23

**Authors:** Lajos Szatmári, Tamás Cserkész, Levente Laczkó, József Lanszki, Cino Pertoldi, Alexei V. Abramov, Morten Elmeros, Barnabás Ottlecz, Zsolt Hegyeli, Gábor Sramkó

**Affiliations:** ^1^ MTA‐DE “Lendület” Evolutionary Phylogenomics Research Group Debrecen Hungary; ^2^ Department of Botany University of Debrecen Debrecen Hungary; ^3^ Department of Zoology Hungarian Natural History Museum Budapest Hungary; ^4^ Bükk Mammalogical Society Eger Hungary; ^5^ Carnivore Ecology Research Group Szent István University, Kaposvár Campus Kaposvár Hungary; ^6^ Department of Chemistry and Bioscience Aalborg University Aalborg Øst Denmark; ^7^ Aalborg Zoo Aalborg Denmark; ^8^ Zoological Institute Russian Academy of Sciences Saint Petersburg Russia; ^9^ Department of Bioscience ‐ Wildlife Ecology Aarhus University Rønde Denmark; ^10^ Milvus Group Bird and Nature Protection Association Tîrgu Mureș Romania

**Keywords:** admixture, backcrossing, conservation genomics, F1 hybrid, genetic diversity, RADseq

## Abstract

Introgressive hybridization can pose a serious threat to endangered species which have an overlapping distribution such as in the case of two polecat species, *Mustela eversmanii* and *M. putorius*, in Europe. The population size of steppe polecat is known to continuously shrink, whereas its sister species, the European polecat, is still somehow widespread. In this study, we perform an analysis using microsatellite (SSR) and genomic (SNP) data sets to identify natural hybrids between polecats. Four populations were genotyped for eight polymorphic SSR loci, and thousands of unlinked SNPs were generated using a reduced‐representation sequencing approach, RADseq, to characterize the genetic make‐up of allopatric populations and to identify hybrids in the sympatric area. We applied standard population genetic analyses to characterize the populations based on their SSR allelic frequency. Only a single sample out of 48 sympatric samples showed exact intermediacy that we identified as an F1 hybrid. Additionally, one specimen was indicated in the genomic data sets as backcrossed. Other backcrosses, indicated by SSRs, were not validated by SNPs, which highlights the higher efficacy of the genomic method to identify backcrossed individuals. The low frequency of hybridization suggests that the difference in habitat preference of the two species may act as a barrier to admixture. Therefore, it is apparently unlikely that polecat populations are threatened by significant introgression. The two species showed a clear genetic differentiation using both techniques. We found higher genetic diversity values in the sympatric steppe polecat population than in the other studies on polecat populations. Although *M. putorius* is a hunted species in most countries, genetic diversity values indicate worse conditions in Europe than in the protected sibling species *M. eversmanii*. Suspending hunting and providing protected status of the former seems to be reasonable and timely.

## 1 INTRODUCTION

The growing availability of massive parallel sequencing and reduced‐representation genomic approaches (e.g. different versions of genotype‐by‐sequencing methods, see Andrews et al., 2016) provides a viable alternative to microsatellite‐based genotyping in conservation genetics of non‐model organisms (Allendorf, 2017). Several studies have demonstrated the comparatively better performance of genome‐wide reduced‐representation genotype data over microsatellites at least when a large number of genomic loci was used (Camacho‐Sanchez et al., 2020; Sunde et al., 2020; Zimmerman et al., 2020). The genome‐wide sampling of high‐density genetic markers can provide an improved resolution for the detection of hybrids and introgressed (backcrossed) individuals (McFarlane & Pemberton, 2019) with some notable exceptions (Ottenburghs, 2021) including polyploidy, large genome sizes and extreme demographic history. Apart from these cases, reduced‐representation approaches may better detect hybridization and—especially—subsequent backcrossing than microsatellites, but only a few recent studies have directly compared (i.e. including the very same individuals in analyses using both markers) the two marker types (e.g. McFarlane et al., 2020) to assess the power of microsatellites and reduced‐representation approaches in detection of hybrid and backcrossed specimens.

Hybridization is an important factor in speciation and evolution (Barton & Hewitt, 1985; Payseur & Rieseberg, 2016), but it also causes concern for conservation biology as it can be a serious threatening factor to several endangered species via introgression (e.g. Allendorf et al., 2001; Bohling, 2016; Grabenstein & Taylor, 2018). Populations can also decline whether hybridization results in unviable or infertile offspring (Allendorf & Luikart, 2007). Moreover, direct or indirect human activities can increase the frequency of hybridization, and such anthropogenic hybridization can even threaten the survival of endangered species (Casas et al., 2012; Daniels & Corbett, 2003; Dierking et al., 2014; Lorenzo et al., 2020; McOrist & Kitchener, 1996; Oliveira et al., 2015; Ottenburghs, 2021). One of the best‐known examples among carnivores is the case of the European wildcat (*Felis silvestris*). Native wildcat populations are imposed to genetic erosion via introgressive hybridization with domestic cats (*Felis catus*) (Daniels & Corbett, 2003; McOrist & Kitchener, 1996; Oliveira et al., 2015). Interbreeding between wildcats and domestic cats and introgression of alien haplotypes from domestic cats into the gene pool of the European wildcat at regionally varying degree are reported in several parts of the distribution area (Hertwig et al., 2009; Tiesmeyer et al., 2020).

Hybridization has been recorded among mustelids (e.g. Cabria et al., 2011; Morris et al., 2020). In the case of the steppe polecat (*Mustela eversmanii* Lesson, 1827), hybridization with the European polecat (*M. putorius* L., 1758) was mentioned among potential threatening factors (Šálek et al., 2013). However, there is no solid evidence in support of continuing hybridization between the two species in the wild. A recent study examined the craniometric and phenotypic diversity of polecats (Cserkész et al., 2021), which indicated several morphologically hybrid individuals. Hybrid identification is based on mixture of parental traits in phenotype and size, but this could be misleading owing to considerable overlap in morphological traits between the two species. In such cases, correct hybrid detection by phenotype may be seriously hindered and hybridization rates might be underestimated (Rhymer & Simberloff, 1996)—undetected hybrids can also hide in sympatric populations due to the wide overlap in phenotypes of the species. Considering that phenotypic hybrid identification can be difficult and unreliable in certain cases, it is crucial to use methods that facilitate this process in order to be able to assess the real extent of hybridization and introgression. The utilization of microsatellite markers showing fixed differences between species has recently been employed in several mammalian studies for hybrid detection (e.g. Beugin et al., 2017; McFarlane et al., 2020). There are also several studies with microsatellites to distinguish between *Mustela* species (Cabria et al., 2007; Fleming et al., 1999; Zhao et al., 2018), but there exists only one single study aimed at exploring the dynamics of hybridization (Cabria et al., 2011). The only published hybridization study in mustelids using a genomic approach examined admixture between divergent lineages of stoat (*M. erminea*) in a phylogeographic study (Colella et al., 2018).

As there is no solid genetic evidence to support continuing hybridization between *M. eversmanii* and *M. putorius* in the wild, hybridization has been assumed on the basis of intermediate fur colouration and cranial anatomy. According to Heptner (1964), the hybridization of polecats was not unusual in the former Soviet Union; however, it was estimated to be of far lesser magnitude than one might have expected from their sympatry and close phylogenetic proximity. Although he reported sporadic hybrids from several regions in Eastern Europe, hybrid swarms were not observed and a zone of introgression between the two species was apparently absent (Heptner et al., 1967; Tatarinov, 1956). Nevertheless, a postzygotic barrier does not exist between the two species as they can be successfully crossbred in captivity; offspring are viable and fertile (Ternovsky, 1977).

**FIGURE 1:**
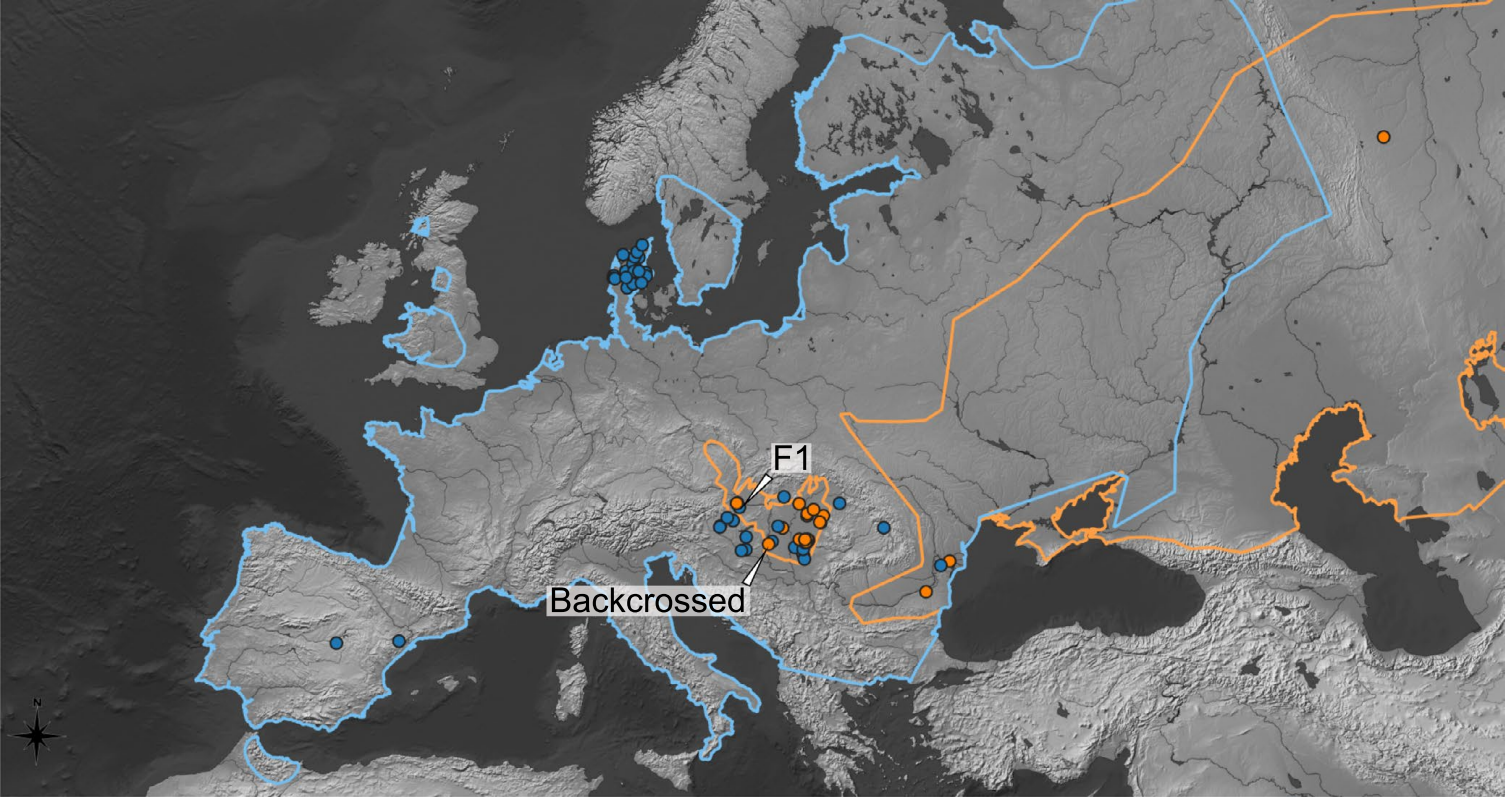
Geographical origin of the samples. Symbols used: orange dots —*Mustela eversmanii*, blue dots—*Mustela putorius*. Central‐Eastern European distribution ranges of *M. eversmanii* (orange line) were redrawn from the unpublished data set of the authors and Hegyeli et al. (2019), Šálek et al. (2013), based on Maran et al. (2016). For *M. putorius* (blue line), we follow Skumatov et al. (2016) and Heptner et al. (1967). Sampling locations of the hybrid and backcrossed individuals are highlighted on the map

The lack of a postzygotic barrier between European polecats and steppe polecats can also be associated with the shallow evolutionary divergence between them; within the Mustelinae subfamily, they are thought to have diverged recently (Law et al., 2018). According to most phylogenetic studies (Koepfli et al., 2008, 2018; Kurose et al., 2000; Law et al., 2018; Sato et al., 2012), *M. lutreola* is the closest relative of the polecats (subgenus *Putorius*: *M. putorius*, *M. eversmanii*, *M. nigripes*), having separated from them approximately 2.13 million years (MY) ago (median time: 1.21 MY) (Kumar et al., 2017). The North American *M. nigripes* is the basal species within the subgenus, and the two Eurasian polecats form a crown group. According to estimates put forward in eight studies, the divergence of the crown group could have taken place 1.2 MY ago (median time: 0.57 MY) (Kumar et al., 2017). Roughly, the same divergence time estimates were most recently published for the separation of the species of subgenus *Putorius* by Hassanin et al. (2021) based on sequences of the whole mitochondrion: 1.1 Mya was estimated for the split off of *M. nigripes* and 0.6–0.7 Mya for the two other species. Since this event, the European species have established a largely allopatric distribution: *M. putorius* is the species of northern and western Europe mainly in forested areas, whereas *M. eversmanii* inhabits grasslands of Eastern Europe and Central Asia. The modern range of *M. putorius* extends from Great Britain to the Ural Mountains (Croose et al., 2018) (Figure 1). The feral range of the domestic ferret *M. furo*, which is the domesticated form of *M. putorius*, includes some areas within European polecat's native range (e.g. the British Isles) and some outside of it, such as New Zealand (Skumatov et al., 2016). European populations of *M. eversmanii* are classified into two subspecies; the range of *M*. *e*. *eversmanii* includes NE Bulgaria, S and E Romania, SE Poland, Moldova, Ukraine east of the Carpathians and southern European Russia (Maran et al., 2016), whereas *M*. *e*. *hungarica* largely inhabits the Pannonian Basin (i.e. SE part of the Czech Republic, SW Ukraine, E Austria, Hungary, N Serbia, S Slovakia and W Romania), where its distribution overlaps with *M. putorius*. A candidate‐gene approach (i.e. the utilization of one or a few highly informative genes) is sometimes suboptimal for understanding of phylogenetic relationships in the Order Carnivora. For example, cytochrome‐oxidase B is one of the most heavily utilized phylogenetic markers phylogenetic reconstruction, but it did not provide significant phylogenetic resolution between *M. eversmanii* and *M. putorius* (Kurose et al., 2000, 2008). Approximately 30 genic regions were used to establish the sister relationship between the above two species (Law et al., 2018); however, the relationship remained partially unsupported (i.e. the maximum‐likelihood bootstrap value remained 65%).

Because of the lack of phylogenetic resolution, the first study to apply a genetic method (Davison et al., 1999) suggested using microsatellites for the study of hybridization and phylogeny of polecats. Wisely et al. (2002) published the first results on the population genetic structure of *Mustela putorius*, *M. eversmanii dauricus* and *M. nigripes* using microsatellite markers, which were followed by subsequent studies (Ciofi et al., 2012; Costa et al., 2012; Møller et al., 2004; Pertoldi et al., 2006). These contemporary studies noted comparable genetic diversity (*H*_E_ = 0.4–0.64) in the analysed European polecat populations, which is similar to that found in other mustelid species (e.g. Colli et al., 2011; Nagai et al., 2012) and is slightly lower than in carnivorans in general (e.g. Eckert et al., 2010; Tashima et al., 2010; Wultsch et al., 2014). Cabria et al. (2011) analysed a data set of 13 microsatellite nuclear markers to characterize continuing hybridization and genetic introgression between European mink (*M. lutreola*) and *M. putorius*. They found that hybridization and genetic introgression occurred at low levels and in an asymmetric way, where only pure European polecat males mate with pure European mink females. Furthermore, backcrossing and genetic introgression were detected only from first‐generation (F_1_) female hybrids of European minks to polecats. They suggested that hybridization and genetic introgression between the two species should be considered a rather uncommon event, although current low population densities of the European mink might change this trend. As no similar study exists for polecats, the study of hybridization between them is therefore timely (see Hassain et al., 2021).

Like *M. lutreola*, *M. eversmanii* was also driven to the brink of extinction in Europe. Thoughtless eradication campaigns against the principal prey species of *M. eversmanii*, the common vole (*Microtus arvalis*) and the European hamster (*Cricetus cricetus*), had a major impact on the population of both polecat species in the 20th century (Šálek et al., 2013). The range and size of populations were drastically reduced with local extinctions, particularly in the Ukraine, where this species is likely to become extinct in the near future (Cserkész et al., 2021; Selyunina, 2017). The European hamster is now a protected species in the European Union (since 2008 in Hungary), and the use of rodenticides is constrained and regulated by the EU Biocides Regulation 528/2012 since 09/01 2013. As a result, hamsters have started to recover in the last decade (remarkably from 2015) and recolonized croplands throughout their former range in the Pannonian Basin. Population sizes of the Pannonian steppe polecat followed this expansion (Cserkész et al., 2020). In contrast, the population of *M. putorius* is believed to be in decline in several European countries (Croose et al., 2018) and is possibly being replaced in some areas around human settlements by beech martens (*Martes foina*) (Skumatov et al., 2016).

However, the recovery of *M. eversmanii* in the Pannonian Basin may be hindered by introgressive hybridization with *M. putorius*. In this study, we employ microsatellites and a reduced‐representation genomic approach, restriction site associated DNA sequencing (RADseq), to assess the extent of hybridization between *M. eversmanii* and *M. putorius* in the Pannonian Basin. We compare our genetic results with allopatric populations of both species. Furthermore, we aim to contribute towards a better understanding of the evolution and conservation of the biological consequences, if any, of hybridization in these mustelids.

## 2 MATERIALS AND METHODS

### 2.1 Sampling

Since the distribution area of the *M. putorius* and *M. eversmanii* partially overlaps, the microsatellite data collection focused on three regions. Firstly, we sampled the Pannonian Basin for both species (*M. eversmanii n* = 31, *M. putorius n* = 17), which is the exclusive distribution area of the subspecies *M. eversmanii hungarica*. Secondly, we also sampled the allopatric area of both species: *M. putorius* was collected in Jutland, Denmark (*n* = 24), allopatric *M. eversmanii* samples originated from the Ural Mts., from which only four samples were available. Microsatellite data set included 75 specimens altogether (Table 1). Although this sampling represents a relatively small part of the distribution ranges (Figure 1), the Pannonian Basin is still a good candidate area for studying natural hybridization between the two species as this part—on the edge of the distribution of *M. eversmanii*—is a potential region where hybridization can be more frequent due to the marginal status of the latter species (Swenson & Howard, 2005). Therefore, we might correctly extrapolate to the whole sympatric area from this territory.

**TABLE 1:** The sample sizes of the different groups of European mustelid species analysed in this study

Species	Distribution	County of origin	Number of samples		Name code
			microsatellites	RADseq	
*Mustela putorius*	Allopatric	Spain	0	2	—
	Allopatric	Denmark	23	2	DanPut
	Sympatric	Hungary, Romania	16	8	PanPut
*Mustela eversmanii*	Sympatric	Hungary, Romania	32	8	PanEver
	Allopatric	Russia	4	2	RusEver

For the genomic analysis, we slightly expanded the geographical coverage of our samples: two samples of *M. putorius* were added from Spain and three samples of *M*. *e*. *eversmanii* were added from the sympatric area in Dobrogea (SE Romania). All samples suspected to be of hybrid origin in the microsatellite analysis were included in the genomic sample set. In addition, we aimed to represent the allopatric area with two specimens from each sampled population. Finally, 10 further samples were chosen randomly from the sympatric area (*M. eversmanii n* = 5, *M. putorius n* = 5). Thus, a total of 22 samples was genotyped using RADseq (Table 1).

Almost all samples, mainly tissue samples from roadkill animals, were collected during a three‐year period between 2017 and 2019. Sampling and monitoring roadkill is a reliable source of information in various cases of ecological and population genetic studies (Schwartz et al., 2020) and has already been used in studies dealing with polecat species (Davison et al., 1999). All our roadkill samples allowed unequivocal taxonomic identification based on phenotypic characters at the species level. All Russian samples were collected by hunters.

Total genomic DNA (gDNA) was isolated from muscle, ear tip or hair samples and stored individually in 96% ethanol or silica gel. DNA extraction followed the protocol described in detail in Cserkész et al. (2015). In short, the tissue was placed in lysis buffer with proteinase K (Thermo Fisher Scientific Ltd.) and incubated overnight at 55°C. Proteins were removed by adding ammonium acetate followed by isopropanol precipitation of gDNA, which was pelletized in a further step by heavy centrifuging. The pellet was washed in 70% ethanol and—after the evaporation of residual ethanol—was resuspended in ultra‐pure water.

### 2.2 Microsatellite data generation

We generated genetic information in two steps. First, we performed a traditional microsatellite (SSR) analysis, to facilitate the comparison of our results with previous studies. Additionally, we used a reduced‐representation genomic approach, restriction site associated DNA sequencing (RADseq), to create a set of high‐density markers (single nucleotide polymorphism: SNP) based on a subset of samples, which was selected in the light of the results of the microsatellite analysis.

We selected microsatellite regions from the literature by choosing those loci which yielded the highest number of alleles in the analysis of Wisely et al. (2002). Of the 12 tested microsatellite loci, one was originally developed for the American black bear, *Ursus americanus* (G1A) (Paetkau et al., 1995), five for the American mink, *Neovison vison* (Mvi057, Mvi087, Mvi232, Mvi111 and Mvi114) (O'Connell et al., 1996), four for the stoat, *Mustela erminea* (Mer005, Mer009, Mvis022, Mvis072, Mer022) (Fleming et al., 1999) and the last one was developed for the closely related black‐footed ferret, *Mustela nigripes* (Mer049) (Wisely et al., 2002). Despite the fact that we tested these loci in gradient polymerase chain reactions (PCR) combined with various PCR mixtures, four of them (Mer049, Mvi057, Mvi114 and Mer022) did not yield PCR products.

Microsatellite loci were amplified in a total volume of 10 μl using 0.05 U DreamTaq DNA Polymerase, 2× DreamTaq Green Buffer, 0.2 mM of each dNTP, 1mg/ml BSA (all reagents from Thermo Fisher Scientific Ltd.), 10 µM of each primer (of which the forward was fluorescent labelled by Thermo Fisher Scientific Ltd.) and 1μl gDNA diluted to 10ng/μl. The PCR conditions were as follows: initial 95°C for 3 min, then 40 cycles of 94°C for 15 s, 54°C (Mvis072, G1A, Mvis232, Mvi111, Mvis087) or 60°C (Mer009, Mvis022, Mer005) for 30 s, 72°C for 30 s and a terminal elongation at 72°C for 14 min in an Mastercycler Gradient PCR Machine (Eppendorf AG). PCR products were capillary electrophoretized on an ABI 3130 Genetic Analyzer (Applied Biosystems) with the use of LIZ‐600 standard. Fragment lengths were determined by the visual inspection of the raw electropherograms by the same person using Peak Scanner v. 1.0 software (Applied Biosystems).

### 2.3 Microsatellite population genetic analyses

We calculated basic population genetic parameters, such as allelic patterns (including private alleles for taxonomic and geographical groups) and observed (*H*_O_) and expected (*H*_E_) heterozygosities with GenAlEx v.6.5 (Peakall & Smouse, 2006). Linkage disequilibrium between pairs of microsatellite loci was evaluated using Genepop v.4.0 (Raymond & Rousset, 1995). Deviations from Hardy–Weinberg equilibrium (HWE), as well as the presence of null alleles, were tested with MicroChecker v.2.2.3 (Van Oosterhout et al., 2004) using default settings. To eliminate the effect of null alleles on the outcome of the population genetic analyses, we used FreeNA (Chapuis & Estoup, 2007) to calculate allele frequencies corrected with the ENA method. *F*_ST_ statistics were calculated to estimate genetic differentiation (Weir & Cockerham, 1984), which was also computed with FreeNA. The inbreeding coefficient (*F*_IS_) was calculated using GENETIX v.4.05.2. (Belkhir et al., 1996), and the corresponding significance levels were estimated after 1000 permutations. To explore potential bottleneck events, our data set was analysed with BottleNeck v.1.2.02. (Piry et al., 1999). Genotype accumulation curves, which we calculated with poppr v.2.8.1 (Kamvar et al., 2014), are often used to determine the minimum number of loci necessary to discriminate between individuals in a population. Since our sample sizes from different regions were markedly different, we performed a rarefaction‐based estimation of allelic richness with ADZE v.1.0 software (Szpiech et al., 2008). With Past v.3.04 software (Hammer et al., 2001), we built an UPGMA tree representing the genetic relationships between the *a priori* morphological groupings of the samples. The calculated topology was based on corrected Cavalli‐Sforza chord distance values which were computed with FreeNA, allowing the software to execute 2000 bootstrap resampling over the loci. Since we were looking for outliers, we decided to visualize the relative position of the individual samples in the genetic space by calculating a principal coordinate analysis (PCoA) with GenAlEx v.6.5.

In admixture analyses, establishing a threshold for admixture coefficient (*Q*) is a widely used method to identify hybrid individuals. In general, there is a trade‐off for the determination of a threshold between type I (i.e. the mistaken assignment of a parental species individual as a hybrid) and type II (i.e. the assignment of hybrid individuals as parental types) errors (McFarlane & Pemberton, 2019). In other words, a researcher should choose between accuracy (type I) and efficiency (type II). Regardless of the aim of a study, it is clear that achieving high efficiency is more feasible than achieving high accuracy (Vähä & Primmer, 2006). In this regard, a relatively low threshold value of 0.1 (10%) proved to be highly effective in former studies (van Wyk et al., 2017). Therefore, we used 0.1 (10%) assignment probability as a threshold to discriminate between the purebred and the admixed groups.

parallelstructure v. 2.3.4 (Besnier & Glover, 2013; Pritchard et al., 2000) software was used to perform a Bayesian *a posteriori* clustering analysis of the microsatellite data set. For computational purposes, we used the resources available from the CIPRES Science Gateway (Miller et al., 2010). The parameter settings of our simulation were 100,000 ‘burn‐in’ steps followed by 500,000 MCMC iterations with five replicates for each *K* value (i.e. the number of genetic groups in our samples) values from 1 to 20. The preferred ancestry model was the admixture model, since it allows each individual to draw some fraction of its genome from each population at any given *K*. The optimal *K* value was estimated after the simulation with the online software Structure Selector (Li & Liu, 2018) based on the Puechmaille method (Puechmaille, 2016), since it outperforms the otherwise widely used method of Evanno et al. (2005) in the case of clearly uneven population sample sizes. To further investigate the possible structure of our data set, we also performed a Tracy–Widom (TW) test, which is a completely different, but an equally efficient method, to estimate the possible number of clusters (Patterson et al., 2006). We used the LEA R‐package (Frichot & François, 2015) to calculate the TW test.

To get more detailed information on the structure of the microsatellite data set, we performed a discriminant analysis for principal components (DAPC) from package adegenet v. 2.0.1 (Jombart, 2008; Jombart & Ahmed, 2011) in R. DAPC analysis partition genetic variation into between‐group and within‐group components to identify groups for which the within‐group component of variation is minimized. We chose the DAPC method since it generally performs better than structure at characterizing population subdivision (Jombart et al., 2010). The number of principal components retained for DAPC was obtained by cross‐validation that resulted in the greatest power of discrimination but avoided overfitting (Figure S1).

Finally, we performed a Bayesian assignment test of samples into discrete hybrid categories using NewHybrids v.1.1 (Anderson & Thompson, 2002). We ran the software with both the Jeffreys prior that weights minor alleles with low frequency and also the uniform prior. In all runs, allopatric specimens were categorized to an *a priori* parental population (Table 1) with their exclusion from admixture (option ‘z+s’), and we ran the simulation for 500k generations with 100k ‘burn‐in’.

### 2.4 Genomic data generation

The gDNA was also used to generate the RADseq data set. We only used high‐molecular‐weight and unfragmented samples which were checked on 1.5% agarose gel. High‐quality samples were measured for their exact double‐stranded (ds) DNA quantity by a Qubit v.3 fluorimeter (Thermo Fisher Scientific) using the Qubit dsDNA HS Assay (Thermo Fisher Scientific), and 210 ng dsDNA was used in an original RADseq library preparation (Baird et al., 2008). In this procedure, we used the rare‐cutting enzyme SbfI‐HF (New England Biolabs, Ipswich, MA, USA) to restriction digest the dsDNA in our samples and ligate custom‐modified Illumina True‐seq P1 adapters to the overhangs generated. These fragments were sonicated in a Bioruptor Pico machine (Diagenode SA.) for five cycles of ultrasonic shearing at 30‐second ‘on’ and 30‐second ‘off’. After a size‐selection step on both sides using SPRI Select Beads Kit (Beckman Coulter Inc.), we ligated custom‐modified P2 adaptors to the fragments by using T4 ligase (New England Biolabs). Two sub‐libraries, each with 12 samples in them, were mixed equimolarly, and a modest PCR amplification of fragments with a P1 and P2 adaptor at their ends was carried out using the High‐Fidelity PCR Master Mix with HF Buffer (New England Biolabs). To minimize PCR artefact at this step, we decreased the number of PCR cycles compared with the original protocol (Baird et al., 2008) and only used 14 cycles of PCR amplification with eight replications, which were pooled into the same library. This was again size‐selected using SPRI beads on both sides, and finally, the size distribution and concentration of our library was checked on a Bioanalyzer machine (Agilent Technologies Inc). The final library was sequenced on an Illumina HiSeq platform with paired‐end, 150 base‐pair (bp) long reads at Novogene Co. Ltd.

Raw Illumina reads were first quality checked and screened for adapter content using fastp v.0.20.1 (Chen et al., 2018) with default settings. The quality‐filtered reads were demultiplexed using the component process_radtags of the Stacks v.2.2 pipeline (Rochette et al., 2019). Sample‐specific reads were then mapped onto the available draft reference genome of *Mustela furo* (GenBank accession number: NC_020638) (Peng et al., 2014) using BWA v.0.7.12 (Li & Durbin, 2009) with default parameters. Mapped reads were then processed with the ref_map pipeline of the Stacks v.2.2 software, and a single SNP was called from each RAD locus using the population module of Stacks. This SNP set was further filtered with vcftools v.0.1.16 (Danecek et al., 2011) for maximum of 5% missingness (max‐missing = 0.95), minor alleles excluded, if present in less than two individuals (maf = 0.091) and SNPs that are in HWE at *p* > 0.05 across the whole data set. This filtering generated a SNP set for 22 samples that was used in downstream analyses of the genomic structure of European polecats.

### 2.5 Genomic analyses

The unlinked SNP set generated in the previous step was first imported into R (R Core Team, 2014) by vcfR v.1.10 (Knaus & Grünwald, 2017) and analysed for basic quantity and quality measures (Figure S2). Here, we focused on placing our studied individuals into a genetic space based on thousands of unlinked SNPs. Thus, we visualized uncorrected‐p genetic distances between our samples on a split network using the NeighborNet method (Bryant & Moulton, 2004) implemented in SplitsTree4 v.4.16.1 (Huson & Bryant, 2006). In case of reticulation, which we assumed here, networks can much better represent the evolutionary pathways than bifurcating trees (Huson et al., 2010).

For an admixture analysis of the SNP data set, we performed a sparse non‐negative matrix factorization (snmf) from LEA 2.0 package (Frichot & François, 2015) in R. This function provides results very close to Bayesian clustering programs such as structure (François & Durand, 2010), but it is optimized to deal with large genotypic matrices such as those we usually obtain from a genomic analysis. We implemented both build functions from this package, the cross‐validation technique and TW test, to estimate the most probable number of clusters.

In a further test of the result of snmf analysis, we performed a DAPC as a complementary clustering method. The repeated use of DAPC also facilitates the comparison of results obtained from the two different data sets. For the same reason, we also performed analysis with NewHybrids on the SNP data set. We ran the software with default settings by assigning all specimens according to their *a priori* taxonomy (Table 1) to one of the parental categories with the possibility of admixture (option ‘z’). To overcome the computational limitations of NewHybrids, we followed the recommendation of Elliott & Russello (2018) and subsampled the SNP data set. To accomplish this, we firstly screened for *F*_ST_ outliers using BayeScan v.2.1 (Foll & Gaggiotti, 2008) with default settings, excluded the outliers and then selected SNPs with the highest discriminatory power from the remaining SNP set using the gl.nhybrids function of dartR (Gruber et al., 2018). We found it reasonable to run the NewHybrids analysis using different SNP sets (one with the 200, another with the 1000 most discriminatory SNPs) and compared the consistency between the results using Jeffreys or the uniform prior. We ran all simulations for 50k generations with 10k ‘burn‐in’.

## 3 RESULTS

### 3.1 Microsatellite genotyping

Our final microsatellite data matrix consisted of a set of 75 individual genotypes based on eight loci. All but one locus proved to be polymorphic in both species. Only locus Mer009 was monomorphic in the Pannonian *M. putorius* population. The number of alleles per loci ranged from 6 to 12. The mean number of alleles was the highest in the Pannonian *M. eversmanii* population (6.125), while the Russian *M. eversmanii* (4) and the Danish *M. putorius* (4.375) populations showed similarly low numbers. In contrast, mean allele numbers were again somewhat higher in the Pannonian *M. putorius* (5) (Table 2). The number of effective alleles was very similar (between 2.5 and 3.0) in populations from Europe, and only the Russian population exhibited a slightly higher (3.4) value, which is most probably the result of the much smaller sample size. The relatively moderate discrepancy between ‘the average number of alleles’ and ‘the effective number of alleles’ suggests that only a few alleles are present with low frequency in the data set. Linkage disequilibrium between markers was not observed (Genepop 4.0, results not shown). We found putatively diagnostic alleles for both species given the limited geographical coverage of our sampling. Twelve alleles (25.5%) proved to be exclusive for *M. putorius* and 22 alleles (38.6%) for *M. eversmanii*. All four tested populations had a set of private alleles as well. Ten alleles were exclusive to the Pannonian *M. eversmanii* population, whereas the Danish *M. putorius* was the population with the fewest private alleles (2). The frequencies of these private alleles were typically under 0.1. However, the most frequent private allele (Locus G1A – 174 bp) occurs with high frequency (0.45) among Pannonian steppe polecats. Moreover, three other private alleles exceeded 0.25 in this population. Unbiased expected heterozygosity (uH_E_) was relatively high in all populations (mean ± SD = 0.615 ± 0.04). In general, genetic diversity was higher in *M. eversmanii* populations than in *M. putorius*. The genetically least diverse population was the Danish *M. putorius* (Table 2).

**TABLE 2:** Basic population genetic characteristics of the studied populations as calculated in GenAlEx

Population	Na (mean ± SE)	Ne (mean ± SE)	*H*_o_ (mean ± SE)	*H*_E_ (mean ± SE)	uH_E_ (mean ± SE)
DanPut	4.375 ± 0.625	2.507 ± 0.508	0.416 ± 0.095	0.475 ± 0.098	0.485 ± 0.100
PanPut	5.00 ± 0.756	2.857 ± 0.635	0.469 ± 0.099	0.509 ± 0.102	0.525 ± 0.105
PanEver	6.125 ± 0.639	2.98 ± 0.267	0.543 ± 0.057	0.641 ± 0.038	0.652 ± 0.039
RusEver	4.00 ± 0.267	3.421 ± 0.225	0.813 ± 0.063	0.699 ± 0.019	0.799 ± 0.021

Abbreviations: *H*_E_, expected heterozygosity; *H*_O_, observed heterozygosity; Na, private alleles; Ne, No. of effective alleles; uH_E_, unbiased expected heterozygosity.

Allelic rarefaction in ADZE showed that all populations, except the Russian *M. eversmanii*, approximated a plateau (Figure S3). Similarly, genotype accumulation curves showed that the otherwise limited number of loci genotyped in this study described the variation displayed by all populations (Figure S4). However, due to the low sample number of the Russian population, we only show its diversity statistics for the sake of completeness, but do not regard them to be representative for that population.

The test of HWE indicated that locus ‘Mvis087’ significantly deviated from the equilibrium. We assumed that the presence of null alleles could have been the cause of deviation. Testing that with MicroChecker v.2.2.3 revealed that most probably there are null alleles on locus ‘Mvis087’ in three populations. To estimate the effects of the presence of null alleles, we compared the uH_E_ values calculated from our original data set with the one calculated from the corrected allele frequencies (ENA method) calculated with FreeNA. The differences between the two values were zero or <1.5%. On that basis, we decided to analyse all eight loci using the corrected frequency values wherever possible.

The test results of the former dramatic population size changes were slightly controversial. The Wilcoxon tests suggest that the Pannonian *M. eversmanii* population suffered a collapse in the past (supported weakly by the infinite allele model (IAM) and more strongly by the stepwise mutation model (SMM)). In contrast, the mode shift test showed a normal L‐shape in all three cases. The Russian *M. eversmanii* population was not tested due to the low sample size. The inbreeding coefficient (*F*_IS_) of all studied populations was significantly positive (i.e. homozygote excess).

**FIGURE 2:**
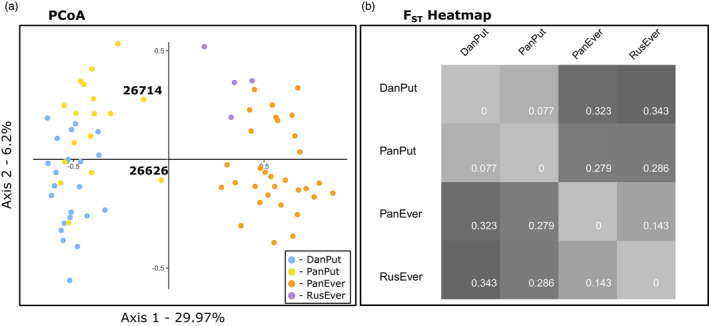
Studied populations’ relative position in the genetic space based on microsatellite data. (a) Principal coordinate analysis (blue dots—*Mustela putorius* from Denmark, yellow dots—*M. putorius* from the Pannonian region, orange dots—*Mustela eversmanii* from the Pannonian region, purple dots—*M. eversmanii* form Russia). (b) Heatmap of pairwise genetic distance based on corrected *F*_ST_.

**FIGURE 3:**
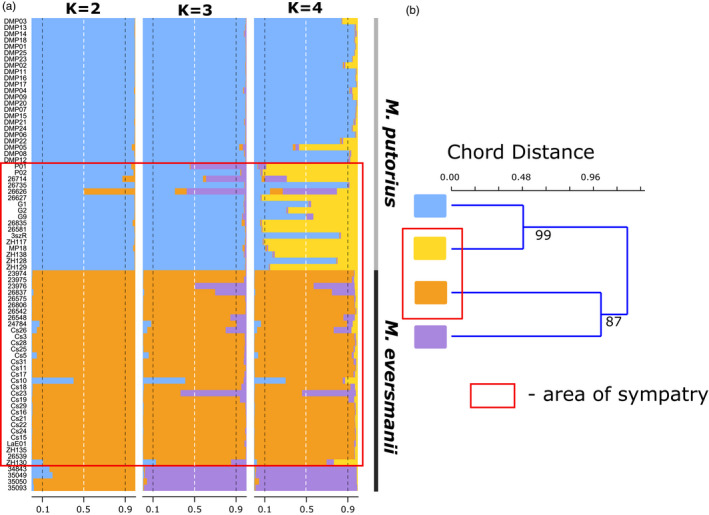
The genetic structure of microsatellite data of the studied *Mustela* populations. (a) structure analysis of microsatellite data at successive numbers of groups. (b) Cavalli‐Sforza chord distance‐based tree of microsatellite allele frequencies with bootstrap support values coming from 1000 bootstrap pseudoreplicates displayed at each corresponding branch (light blue rectangle—*Mustela putorius* from Denmark, yellow rectangle—*M. putorius* from the Pannonian region, orange rectangle—*Mustela eversmanii* from the Pannonian region, purple rectangle—*M. eversmanii* from Russia). The red rectangle represents the sympatric area

We estimated the genetic differentiation of the two species by calculating the F‐statistic (*F*_ST_) based on the corrected allele frequencies (ENA method). The calculated *F*_ST_ = 0.283 is smaller than that reported for *M. putorius* and *M. lutreola* (*F*_ST_ = 0.531) by Cabria et al. (2012). We also calculated the pairwise *F*_ST_ for each population (Figure 2b). The most significant difference in the fixation index was found between the allopatric populations of the two species (RusEver – DanPut *F*_ST_ = 0.343), whereas the sampled populations of *M. putorius* were highly similar (*F*_ST_ = 0.077). The genetic difference between the two populations of *M. eversmanii* (*F*_ST_ = 0.143) was higher, but at the same time smaller than the difference between the two taxa. The corrected Cavalli‐Sforza chord distance‐based tree (Figure 3b) represents the same pattern.

In conformity with the above‐mentioned results, a PCoA separated two sets of individual microsatellite genotypes in full agreement with the *a priori* morphological grouping (Figure 2a) and highlighted the genetic differences between the *M. eversmanii* populations. This ordination also identifies two outliers (#26626 and #26714) from the Pannonian *M. putorius* population, which occupy an intermediate position between the two species (Figure 2a).

We performed a Bayesian clustering (structure) analysis to reveal the genetic structure of our microsatellite data and identify individuals with admixed ancestry. Evanno's method suggested *K* = 2 as the most probable number of groups (Figure S5). Once again the two identified groups were equivalent to the phenotypic species clustering (Figure 3a). At the same time, *K* = 4 grouping, supported by Puechmaille's method and the Tracy–Widom test (Figure S6), represents nearly perfectly the four sampled populations. At the intermediate level of population structure complexity (*K* = 3), the structure software separates the Russian steppe polecat samples from the Pannonian steppe polecat samples. At levels of population complexity higher than *K* = 4, new clusters caused disturbance into previous and biologically highly meaningful structure, so the individual assignment probability values became uncertain (Q<50%) in each sampled population. This means that those clusters are most probably ‘ghost’ or spurious groups (Puechmaille, 2016).

Only three individuals showed significant signs of admixture (individual assignment less than 90%) on each level of probable clustering (*K* = 2–4). Two of them, sample #26626 (Bősárkány, W Hungary) and #26714 (Szombathely, W Hungary), belonged to the *M. putorius* phenotype group. At *K* = 2, when the two species were separated, the group identity for the sample #26626 was equally probable (*Q* = 0.5) for both groups, whereas in sample #26714 the invasive genetic material proportion just exceeded the 10% threshold determined formerly to identify individuals with admixed ancestry. The third putative admixed specimen was sample ‘Cs10’ (*M. eversmanii*, Battonya, SE Hungary). Although this individual was clearly identified as *M. eversmanii* based on phenotypic traits, its SSR genotype resembles Danish *M. putorius*.

**FIGURE 4:**
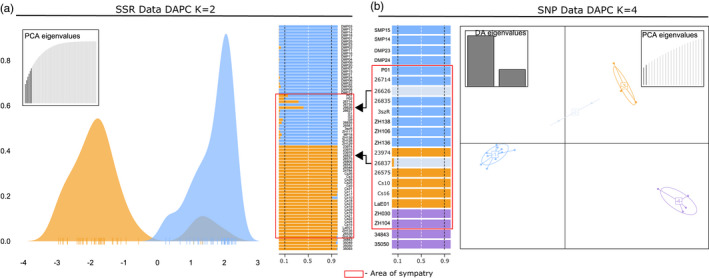
Comparison of DAPC analysis results of microsatellite (a) and SNP data (b). (a) Microsatellite data at *K* = 2 (the most probable number of groups); light blue—*Mustela putorius* samples; orange—*Mustela eversmanii* samples; (b) SNP data at *K* = 4 (the most probable number of groups); light blue—*M. putorius* samples; orange—*M*. *e*. *hungarica* samples; purple—*M*. *e*. *eversmanii* samples; light grey—group of individuals with admixed ancestry. The red rectangle represents the sympatric area. Arrows highlight the hybrid individuals in both data sets

Microsatellite results were also obtained from DAPC analysis. The Bayesian information criterion (BIC) reached a minimum value at 2, suggesting *K* = 2 as the most probable number of clusters (Figure S7). At *K* = 2, individuals were clustered in accordance with the *a priori* external morphological species identification. The two species were divided into two highly distinct clusters. Similar to the microsatellite structure analysis, three specimens’ ancestry proportion exceeded the 10% threshold for both species. However, in addition to the two putative hybrids identified by Bayesian clustering (#26626, #26714), sample ‘P01’ (*M. putorius*, Felsőszölnök, W Hungary) was added as a potential third admixed specimen in this analysis. All three individuals were identified as *M. putorius* based on morphology (Cserkész et al., 2021). All other samples were assigned to one of the clusters with more than 90% probability (Figure 4a).

**FIGURE 5:**
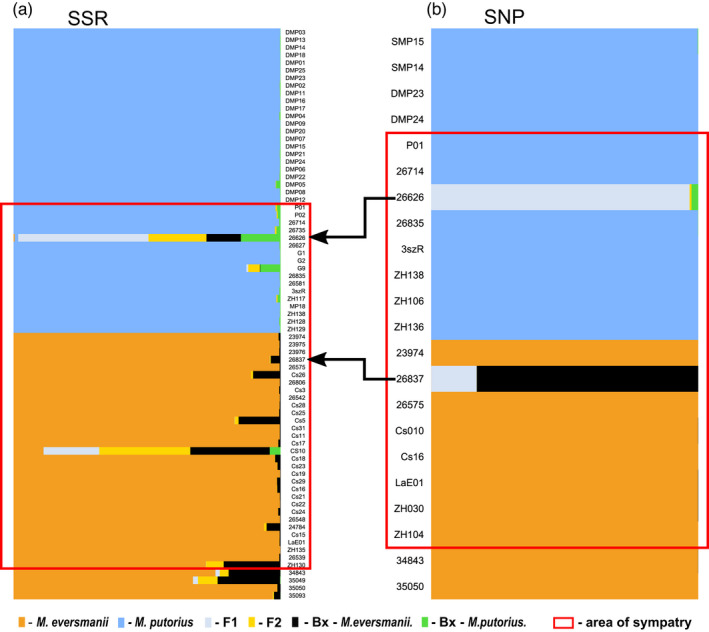
NewHybrids analyses using uniform prior based on (a) microsatellite data and (b) SNP data based on 200 selected SNPs with the highest discriminatory power

Finally, we ran assignment test of hybridity using NewHybrids. The analyses with different priors yielded similar results; therefore, we only present the run using the uniform prior (Figure 5a). The analysis identified two specimens (#26626 and Cs10) with large probability of hybrid origin (i.e. being an F1 or F2 hybrid) and several potential backcrosses mostly towards *M. eversmanii*. None of the potential backcrossed specimens had hybrid probability exceeding 23%.

### 3.2 Genomic results

The raw genomic data set consisted of a total of 177,303,172 paired‐end reads of 150 bp length. After demultiplexing and filtering for low‐quality reads, adapter contamination and ambiguous barcodes with a maximum of one mismatch, we retained a total of 149,386,828 (84.2%) reads. The average paired‐end read count across samples was 7,183,240 (min. 4,145,426; max. 20,169,230).

All reads could be successfully mapped to the reference genome of *Mustela furo*. The ref_map pipeline of stacks did not identify any completely unmapped read. Skipping reads with insufficient mapping qualities (6.3%) and excessively soft‐clipped alignments (6.4%) retained 129,398,212 (87.3%) alignments in total (min. 83%; max. 90.7% across samples). Based on these alignments, 616,542 could be built with a mean coverage of 21× (min. 12.3×; max 60–7×). The mean length of loci was 296.4 bp.

The data set contained 164,644 unlinked SNPs (i.e. a single SNP of each polymorphic locus) in total. Filtering for minor allele frequency, missingness and HWE resulted in 10,433 SNPs retained for all downstream analyses. These SNPs were checked for read depth and genotype quality for each individual (Figure S2). The 1st and 3rd quartiles of read depth across individuals and SNPs fell within the range of 9× (ZH030) – 83× (#26626), with a median ranging from 11× (ZH030) to 64× (#26626) and a mean ranging from 12× (ZH030) to 65× (#26626). The median genotype quality was 40 in all samples, whereas the lowest mean value was 35 (ZH030) and the lowest 1st quartile was 31 (ZH030) (Figure S2a).

**FIGURE 6:**
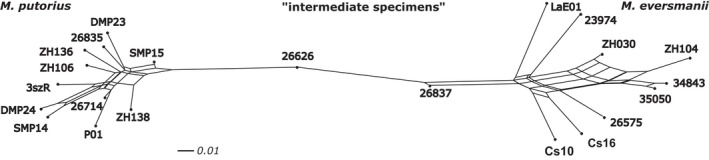
NeighborNet analysis based on 10,433 unlinked SNPs identified from the studied *Mustela* samples

The patterns of NeighborNet based on the SNP data set support the findings of the SSR analysis, in the sense that the two species are clearly separated with no reticulation between them (Figure 6). *M. eversmanii* samples are more distant from each other than the *M. putorius* samples, and a clear edge separates the samples collected east of the Carpathians from other *M. eversmanii* specimens. The intermediate space is occupied by two specimens (#26626, #26837), suggesting that they are the ones with admixed ancestry. Both specimens are situated closer to the species to which they were classified by pelage (Figure 6).

**FIGURE 7:**
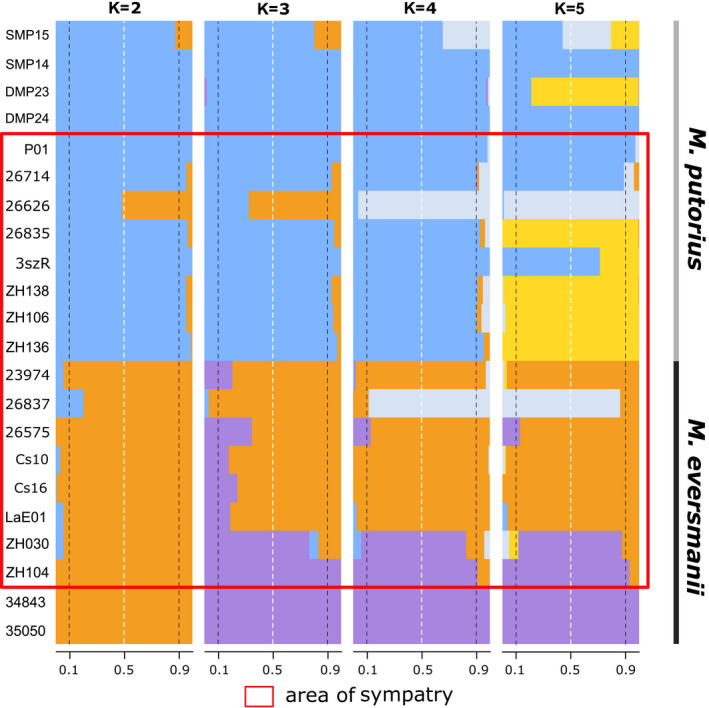
Co‐ancestry‐based sparse non‐negative matrix factorization (snmf) analysis of SNP data. (*K* = – number of groups; the rectangle represents the sympatric area)

For the admixture analysis and clustering, we used the snmf method from the LEA package. The cross‐validation algorithm of the snmf function suggested *K* = 2 as the most probable grouping. Conversely, the other cluster number assessment method from the LEA package, the Tracy–Widom test, returned *K* = 4 as the most probable level of complexity. Besides that, the majority of individual admixture coefficients dropped under 90% at *K* = 6, and it kept decreasing as *K*‐values increased further. Therefore, only the results between *K* = 2 and *K* = 5 are presented here (Figure 7). Once again, the differentiation of the two species is evident. The two estimated clusters at *K* = 2 represented the prior species classification, and the mean of the admixture coefficients was far above 95%. As the complexity grows, the clustering pattern follows the hierarchy of microsatellite‐based pairwise *F*_ST_ values among populations, with a shift at *K* = 4. The fourthly introduced cluster was a group of hybrids rather than a ‘real’ population. Only three samples (#26626, #26837 and SMP15) were clustered to the hybrid group (*Q*‐values >0.4), whose ancestry proportions exceeded the 10% threshold from both species at *K* = 2. The #26626 specimen was identified as a hybrid group member with 99% probability. Besides that, the four samples outside the Carpathians (ZH030, ZH104, #35050 and #34843) are clustered together.

An exploratory multivariate analysis of SNPs (DAPC) supported the clusters returned by the snmf method at *K* = 4 with only a few differences (Figure 4b). The four groups could be defined as (I) the *M. eversmanii* samples from east of the Carpathians (II) *M. eversmanii* samples from the Pannonian region (III) all the *M. putorius* samples across the whole sampled area and (IV) the two samples from the Pannonian region with admixed ancestry (#26626, #26837). This time the SMP15 sample from Spain clustered together with other European polecats. The first axis separates the two species. The subspecies of *M. eversmanii* are mainly separated along the second axis. The hybrid group is in an intermediate position (Figure 4b).

Finally, we also ran the assignment of specimens into hybrid categories by NewHybrids based on 200 highly discriminatory SNPs. We found the results based on Jeffreys prior and the uniform prior to be highly concordant, so we only show and interpret results based on the latter (Figure 5b). This analysis clustered all but two individuals to one of the parental populations with more than 99% probability. Of the two samples with mixed ancestry, specimen #26626 was identified as a F1 hybrid (probability 97%), whereas specimen #26837 was assessed to be a backcross to *M. eversmanii* with high certainty (probability 83%).

## 4 DISCUSSION

### 4.1 Population genetic characterization of the polecat populations

**FIGURE 8:**
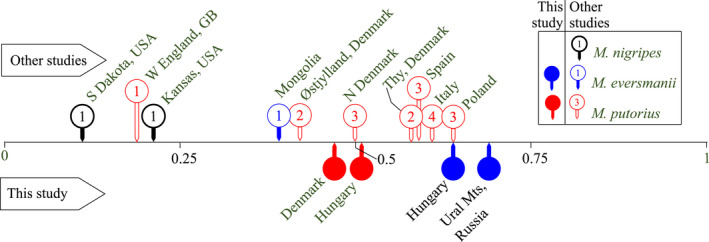
Reported heterozygosity values (*H*_E_) from studied polecat populations. Results of this study are shown under the line, and published results are presented above the line, such as 1—Wisely et al. (2002), 2—Møller et al. (2004), 3—Pertoldi et al. (2006), 4—Ciofi et al. (2012). Black markers refer to *M. nigripes*, red ones to *M. putorius* and blue markers to *M. eversmanii*. Solid blue and red markers are the results published in this study

We studied the genetic structure of sympatric and allopatric populations of *M. eversmanii* and *M. putorius*. In this respect, this is the first population genetic study of a European population of *M. eversmanii*. We expected to detect a serious loss of heterozygosity in the Pannonian *M. eversmanii* population, as this population has shown a severe decline in the last century (Ottlecz et al., 2011). In contrast to that, and in concordance with the findings of Wisely et al. (2002), we found higher heterozygosity in the Pannonian *M. eversmanii* population than in *M. putorius* populations (Table 2). Nonetheless, Wisely et al. (2002) estimated much lower heterozygosity for both *M. eversmanii* (H_E_=0.39) and *M. putorius* (*H*_E_ = 0.17); they found worryingly low values for *M. nigripes* populations in Kansas and South Dakota (Figure 8). Although this difference can also come from the usage of different sets of markers, we believe our comparison is still useful. The heterozygosity of the Pannonian *M. eversmanii* population is the highest reported for any wild polecat population studied (see Figure 8). This genetic diversity does not appear to be the result of introgression of alleles from *M. putorius*, as demonstrated by the high number of private alleles found in the sympatric population and our results on admixture (Figures 3 and 4). The higher genetic diversity of our Urals’ population cannot be regarded as directly comparable with the Pannonian population due to the restricted sample size. Similarly, the Inner Mongolian sample of Wisely et al. (2002) (*n* = 5) may also show a biased value. Similarly to Pertoldi et al. (2006) (*H*_E_ = 0.5), our sample of the Danish European polecat population displayed the lowest value of genetic diversity (uH_E_ = 4.85). Conversely, Møller et al. (2004) reported higher heterozygosity (*H*_E_ = 5.83) in the Danish population based on 83 samples and six microsatellite loci. In this perspective, Cabria et al. (2011) described a relatively low (*H*_E_ = 0.357) heterozygosity for *M. putorius* determined from 114 samples across the whole distribution area from Russia to Turkey and Spain. In the meta‐analysis of Garner et al. (2005), the mean genetic diversity of ‘healthy’ mustelid (Mustelidae) populations is the lowest among carnivorans (mean *H*_E_≈0.6). In full concordance, heterozygosity values reported in polecat populations (Figure 8) of around 0.6 are at the high end of this range. Garner et al. (2005) also found the genetic diversity of ‘demographically challenged’ populations to be significantly lower. In this regard, it is not surprising that the heterozygosity of populations, which suffered a serious decline during the last century, is under 0.25 (Figure 8).

A homozygous excess relative to what would be expected under HWE (i.e. more homozygotes than would be expected according to HWE proportions) can be observed as demonstrated by positive inbreeding coefficients (*F*_IS_) in all populations. This can hint at contemporary inbreeding. We detected a former bottleneck event, with equivocal results, only in the Pannonian *M. eversmanii* population. Similarly, Møller et al. (2004) could not detect population fluctuations in Danish *M. putorius* populations. In contrast, the results presented in Pertoldi et al. (2006) suggest that European polecat populations in southern Denmark and Poland have declined and populations from northern Denmark and the Netherlands have expanded recently. The conservation status of the Danish polecat population is assessed as unfavourable on the basis of a declining game bag (Therkildsen et al., 2020). We expected a positive result for the bottleneck test (at least) in the Pannonian *M. eversmanii* population. As the SMM test suggested a bottleneck, which is considered to be the most suitable mutation model for microsatellites (Putman & Carbone, 2014), we are inclined to accept this result. However, the availability of a relatively limited number of SSR loci comes at a cost to bottleneck detection (Piry et al., 1999) so that these results should be treated with caution.

### 4.2 Identifying individuals with admixed ancestry

We focused more on testing contemporary hybridization between two closely related European mustelid species using two different molecular marker systems. Only specimen #26626 showed clear signs of admixed ancestry regardless of the specific analysis. It was localized in an intermediate position both on the PCoA analysis of the microsatellite data set and on the NeighborNet based on SNPs (Figures 4 and 5). Moreover, each time when the implemented clustering methods identified two groups (typically the two species), this specimen's group membership was equally probable for both groups (*Q* = 0.5). Vähä and Primmer (2006) found that the efficient detection of F1 hybrid individuals requires the use of 24 loci at 0.12 *F*_ST_ between hybridizing parental populations, but only 12 loci are sufficient in case of *F*_ST_ = 0.21. If we assume a linear relationship, the eight loci amplified in our study should be sufficient to unequivocally identify hybrids, since *F*_ST_ is 0.283 between the two studied species. By accepting this reasoning, it is safe to conclude that *M. putorius* and *M. eversmanii* do hybridize in the wild. The direct assessment of hybrid origin using NewHybrids clearly corroborates this statement as it classified this individual as an F1 hybrid between the two studied species regardless of the marker system used (Figure 5). Nevertheless, interbreeding appears to be occasional, since only one specimen was identified as an unequivocal F1 hybrid among the 48 sympatric samples (2.08%). The low percentage of F1 hybrids detected in this study is similar to that found between *M. putorius* and *M. lutreola* (2.24%) (Cabria et al., 2011). Surprisingly, specimen #26626, which we identified genetically as a F1 hybrid, was unambiguously classified as *M. putorius* based on the species‐specific morphological traits of this specimen and our morphometric study (Cserkész et al., 2021). This highlights that phenotypic characters could mask the correct identification of F1 hybrid polecats probably as a result of genetically dominant traits, which give a false‐negative phenotype. Such questions could be studied by captive crossing of the two species, which would allow investigation of F2s and backcrosses both morphologically and genetically.

Hybridization is more frequent, as a rule, at the periphery of the distribution area (Swenson & Howard, 2005)—at the very place where individual #26626 was collected in Western Hungary (see Figure 1). At this western edge of the range of *M. eversmanii*, the scarcity of appropriate mates might lead to this hybrid similar to what Cabria et al. (2011) reported for *M. lutreola*.

Since introgression with *M. putorius* was identified as a major threat to *M. eversmanii* (Šálek et al., 2013), the extent of backcrossing has a special conservation importance. From the study of Grafodatsky et al. (1978), we know that the hybrids of *M. eversmanii* and *M. furo* (domestic ferret) are fertile, opening the possibility for introgressive hybridization between our studied sympatric populations. As suggested by the model of Boecklen and Howard (1997), each successive generation of backcrossing erases roughly half of the introgressed genome in backcrossed individuals leading to an exponentially growing number of markers needed to efficiently identify admixed individuals. This can lead to 52% of fourth‐generation backcrosses being undetectable as backcrosses when only 10 (diagnostic) markers are used. By contrast, 1000 diagnostic markers would detect 85% of ninth‐generation backcrosses (McFarlane & Pemberton, 2019). This means that the application of high‐density markers, such as SNP data from RAD sequencing, will provide a deeper insight into the extent of backcrossing (McFarlane et al., 2020; Pilot et al., 2018).

Only one specimen (#26714, Szombathely, W Hungary), from the *M. putorius a priori* phenotypic group, was clustered as a backcrossed individual by all the four analyses based on SSRs (PCoA, structure, DAPC and NewHybrids). However, the genomic approach nested this sample well‐within *M. putorius* (Figure 6). As a relatively low number of SSR indicates the backcrossed nature of this specimen, we accept this indication as a false positive. Similarly, we suspect other indications of backcrosses in the SSR data set (‘P01’, ‘Cs10’), usually by one method, are false positives.

Compared to SSRs, with the analysis of 10,433 unlinked SNPs, we expected the number of backcrossed individuals to increase compared with those identified on the basis of microsatellite data. Surprisingly, if a 10% threshold for admixed origin was taken, none of the implemented SNP‐based ancestry analyses corroborated the findings of the microsatellite‐based results of backcrosses. Instead, all four tests (NeigborNet, DAPC, snmf, NewHybrids) identified only a single individual of *M*. *eversmanii* (#26837, Érsekcsanád, C Hungary) as backcrossed. The morphology, as well as the distribution of the ancestry proportion (LEA *K* = 2), the position on the NeighborNet and the high probability (83%) of being a backcrossed individual in the NewHybrids analysis suggest that specimen #26837 is a result of series of backcrossings with *M. eversmanii*. In agreement with that, it was collected in a barely forested, mainly agricultural lowland region, where the environmental conditions seem to be much suitable for *M. eversmanii*. Unfortunately, this specimen was not included in the morphometric analyses made by Cserkész et al. (2021). Additionally, none of the analyses based on SSRs showed this specimen as backcrossed, which clearly highlights the limitation of a handful of SSRs for detecting backcrosses.

Interestingly, snmf (LEA) analysis clustered the *M. putorius* sample ‘SMP15’ from Spain, outside the sympatric area, to the admixed group. This surprising result is unlikely to be the sign of recent hybridization; instead, it could be a result of inheriting shared ancestral alleles with *M. eversmanii* from *M. lutreola*, since *M. putorius* and *M. lutreola* do hybridize in Spain (Cabria et al., 2011). In accordance with this, NewHybrids did not classify this specimen as a hybrid of any sort.

Clearly, thousands of SNPs can easily outperform microsatellites (e.g. Camacho‐Sanchez et al., 2020; Bradbury et al., 2015; Hoffman et al., 2014; Lemopoulos et al., 2019; Sunde et al., 2020; Zimmerman et al., 2020). Nevertheless, the utility of SSRs in the detection of hybrid specimens in the F1 generation is demonstrated by our results. By using only a handful SSR loci, we were able to confidently detect a F1 hybrid by both marker systems. These results suggest that if the parents belong to two well‐defined species, F1 hybrids can be detected even by the usage of a relatively low number of informative SSR loci.

### 4.3 Genetic differences between the studied polecat taxa

We regard the study of Cabria et al. (2011), who were focusing on hybridization patterns between *M. putorius* and *M. lutreola* using microsatellite data, as an especially useful comparison with our results. As *M. lutreola* is the closest relative of the subgenus *Putorius*, we expect similar patterns but with a greater degree of genetic dissimilarity between the subgenera. Private alleles and allele frequency differences are considered as significant population or species distinction parameters (Cabria et al., 2011; Oliveira et al., 2006). Given this, it is surprising that the patterns of private allele distributions are very similar in the two studies at the level of species. A total of 23 (34.33%) and 20 (27.03%) private alleles were found in European mink and European polecat, respectively. In comparison, we found 22 (38.6%) and 12 (25.5%) private alleles in the steppe polecat and the European polecat, respectively. The phylogenetic relationships between the studied populations correlate nicely with genetic differentiation as measured by the fixation index (Figure 2b). The corrected *F*_ST_ values were 0.283 between *M. putorius* and *M. eversmanii* (allopatric and sympatric samples pooled together), whereas the difference between *M. putorius* and *M. lutreola* was 0.531 (Cabria et al., 2011). Genetic differentiation also correlates with taxonomy within the subgenus *Putorius*; the two described subspecies of *M. eversmanii* differed more (*F*_ST_ = 0.143) than the two sampled populations of *M. putorius* (*F*_ST_ = 0.077). Nevertheless, reaching a solid taxonomic conclusion on the subspecies status of *M. eversmanii hungarica* would require a phylogeographical approach that is beyond the scope of our current study.

### 4.4 Consequences for nature conservation and future perspectives

The shortage of occurrence data suggests that *M. eversmanii* has been driven to the brink of extinction in Central‐Eastern Europe. However, this conclusion is not supported by the population genetic results of this study. The apparent alarming rate of decline of *M. eversmanii* may instead be an indication of the lack of surveys rather than that of the decrease of population sizes. Alternatively, our results may document a rapid population recovery following a precipitous decline of the species. The contrary seems to be the case for *M. putorius*, which is not a protected species in most countries and listed on Annex V of the Habitats Directive—an Annex that largely concerns animals for which hunting or gathering can still be allowed. Our population genetic results hint at *M. putorius* populations probably being in worse condition in Europe. Suspending hunting and providing protected status seems to be reasonable and timely, particularly in Western Europe where the polecat's conservation status is unfavourable (Anonymus, 2021). The species also has unfavourable conservation status in Denmark and in most other Western European countries, while its conservation status in Hungary and elsewhere in Eastern European countries is assessed as favourable. This should be revised in light of our conservation genetics data on Pannonian *M. putorius*.

Our study revealed a low level of hybridization between steppe polecats and European polecats in the Pannonian Basin where the two closely related species live in sympatry. Consequently, hybridization does not currently pose a threat to the sympatric polecat populations studied. However, this could change as ecological changes due to human activity are increasing. The European hamster, one of the most preferred prey of *M. eversmanii*, has tended to invade villages in the Pannonia Basin since 2010 (Cserkész et al., 2020), so it is not surprising that the number of the *M. eversmanii* individuals found inside or close to villages is increasing (our own unpublished data), and this trend is expected to continue. As a result, in villages the possibility of interactions between the two polecat species can be expected to be more frequent in future. Long‐term monitoring is required to acquire samples to track whether hybridization does increase especially in such situation where ecological conditions are likely to change. A good precedent is given by the track record of hybridization between polecat and ferret in the British Isles (Costa et al., 2012; Davison et al., 1999).

## 5 CONCLUSIONS

The genetic diversity of the studied steppe polecat population was surprisingly high and higher than any of the European polecat populations studied, although the latter is a hunted game species and not protected. This practise should be revised in the future. The genomic approach, RADseq, clearly outperformed conventional SSRs in detecting admixed individuals, because backcrossed specimens were inconsistently identified across analyses based on SSRs, whereas SNPs indicated the same pattern regardless of the analysis. However, it was possible to identify an F1 hybrid using a smaller number of informative microsatellite loci as this specimen was consistently detected as an F1 hybrid in all analyses irrespective of the molecular marker system. We only observed hybridization in the Pannonian area at a low frequency (around 2%) and at the periphery of the distribution area of *M. eversamnii*, which clearly shows this sympatric area cannot be regarded as a hybrid zone between these two closely related species. The number of hybrid individuals could be low in Pannonian populations of both *M. eversmanii* and *M. putorius*, but due to changing ecological conditions (e.g. the urbanization of the European hamster) this number can be expected to increase in the future. Further regular genetic monitoring is recommended to see whether introgression will begin to occur.

## CONFLICT OF INTEREST

None declared.

## Supporting information

Supplementary Material

## Data Availability

The raw microsatellite genotypes are available in the Supporting Information. RADseq reads are uploaded to NCBI SRA database under the following BioProject (ID PRJNA751885).
