# Supplementary material for: A comparison of microsatellites and genome‐wide SNPs for the detection of admixture brings the first molecular evidence for hybridization between Mustela eversmanii and M. putorius (Mustelidae, Carnivora)
Source: Evol Appl. 2021 Aug 23;14(9):2286–304. doi: 10.1111/eva.13291 (PMC8477604; doi:10.1111/eva.13291)
Supplement: Supplementary file 1 — Supplementary Material [file EVA-14-2286-s001.pdf]

Supplementary Materials

SUPPLEMENTARY FIGURE 1 Result of cross-validation plot for 10,433 unlinked SNPs in our DAPC analysis using varying numbers of PCs as implemented in R-package adegenet v. 2.0.1 (Jombart, 2008; Jombart & Ahmed, 2011).

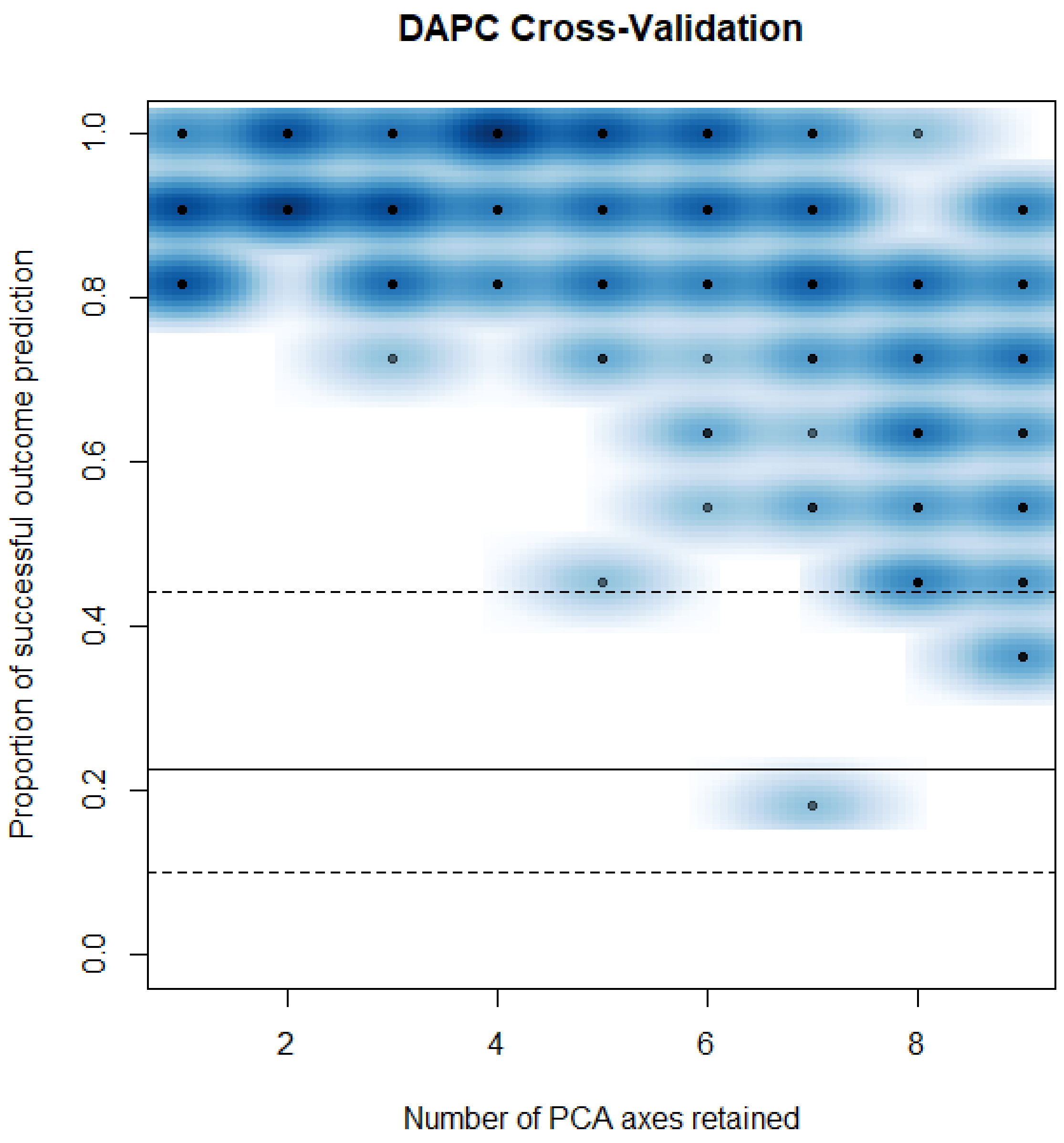

SUPPLEMENTARY FIGURE 2 Basic quantity and quality measures for the 10,433 unlinked SNPs generated in our RADseq experiment and used in downstream genomic analyses. Read depth (A) and genotype quality (B) of each sample included.

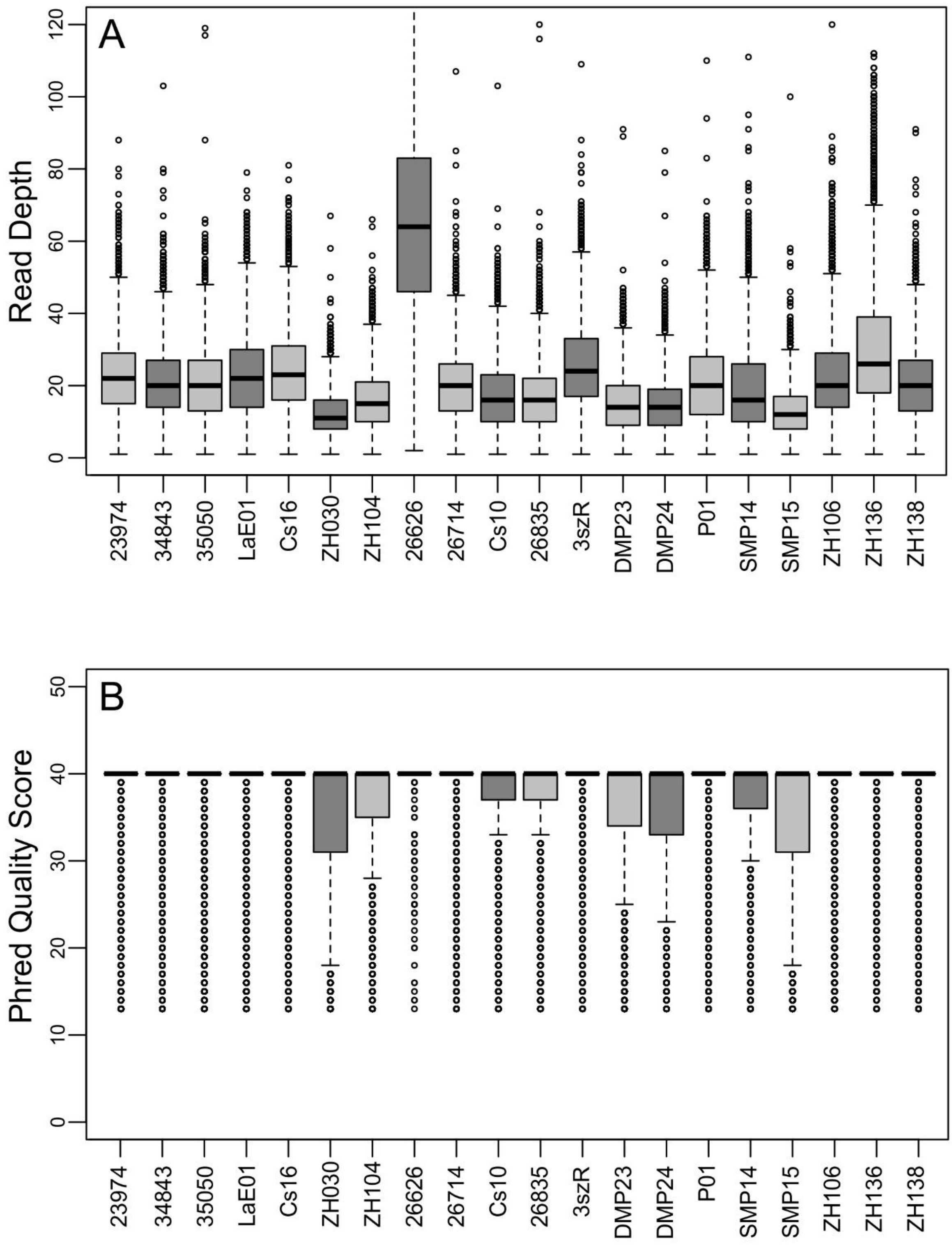

SUPPLEMENTARY FIGURE 3 Allelic rarefaction of microsatellite data for the studied populations. Whiskers represent the standard error at each sample. Population names abbreviated as: PanEver – *Mustela eversmanii*, Hungary and Romania; PanPut – *M. putorius*, Hungary and Romania; DanPut – *M. putorius*, Denmark; RusEver – *M. eversmanii*, Ural Mts., Russia.

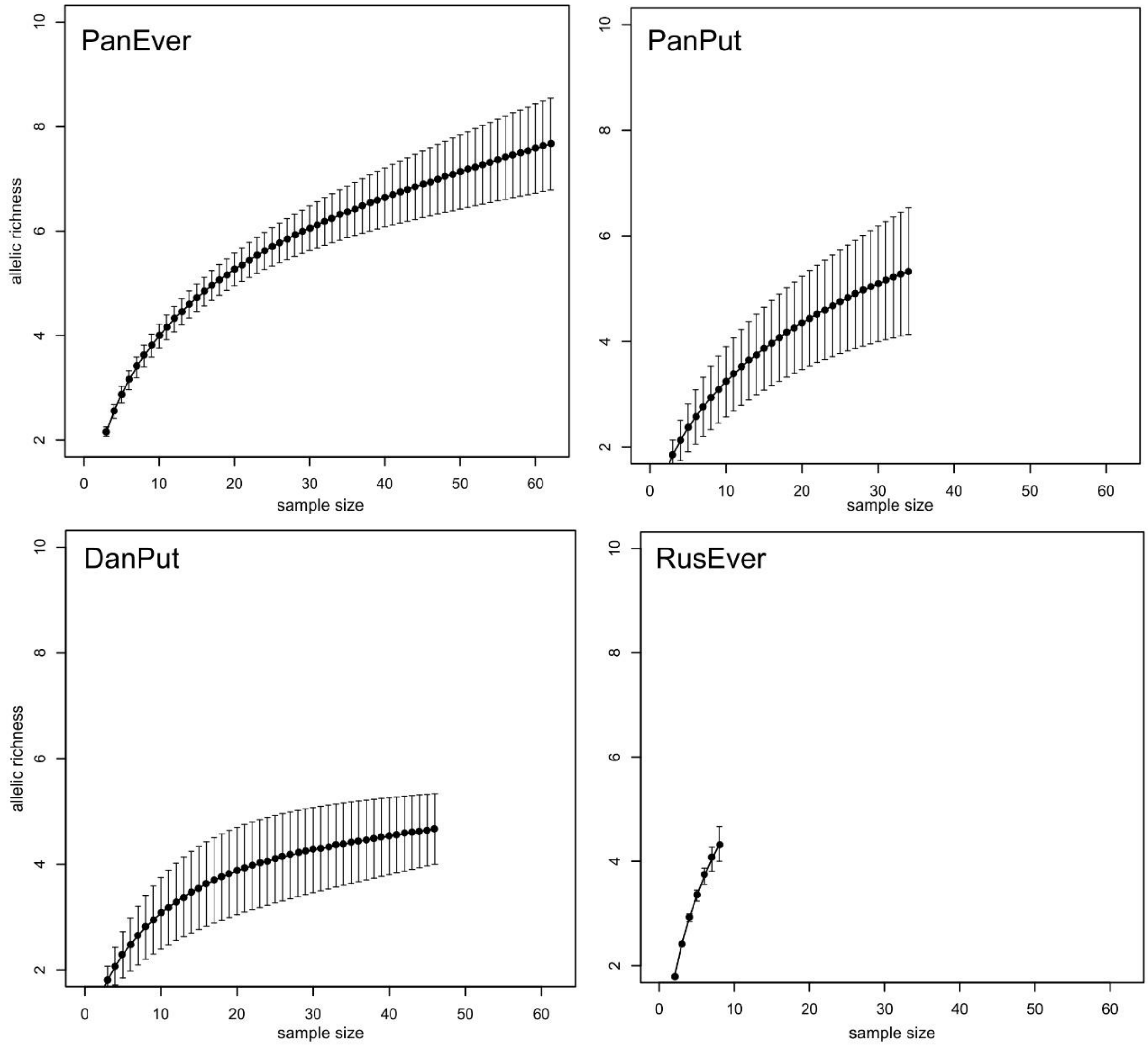

SUPPLEMENTARY FIGURE 4 Multi-locus genotype accumulation curves of microsatellite data for the studied populations. Population names abbreviated as: PanEver – *Mustela eversmanii*, Hungary and Romania; PanPut – *M. putorius*, Hungary and Romania; DanPut – *M. putorius*, Denmark; RusEver – *M. eversmanii*, Ural Mts., Russia.

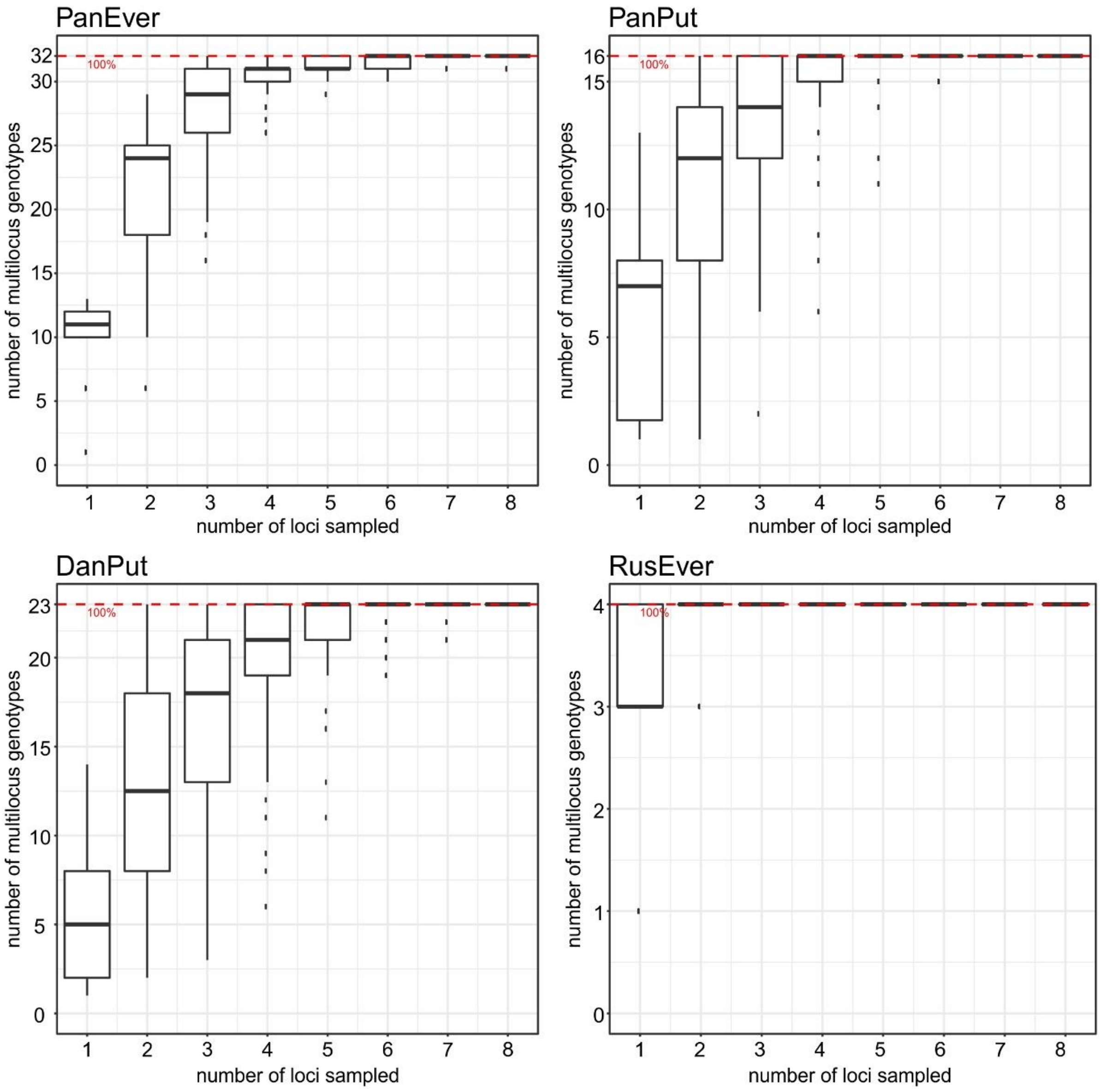

SUPPLEMENTARY FIGURE 5 Most probable number of clusters (K) as suggested by the method of Evanno (Evanno et al. 2005) and value of Ln Pr(X|K) and ΔK according to the suggestion of Janes et al. (2017) as implemented in Structure Selector (ttps://lmme.ac.cn/StructureSelector).

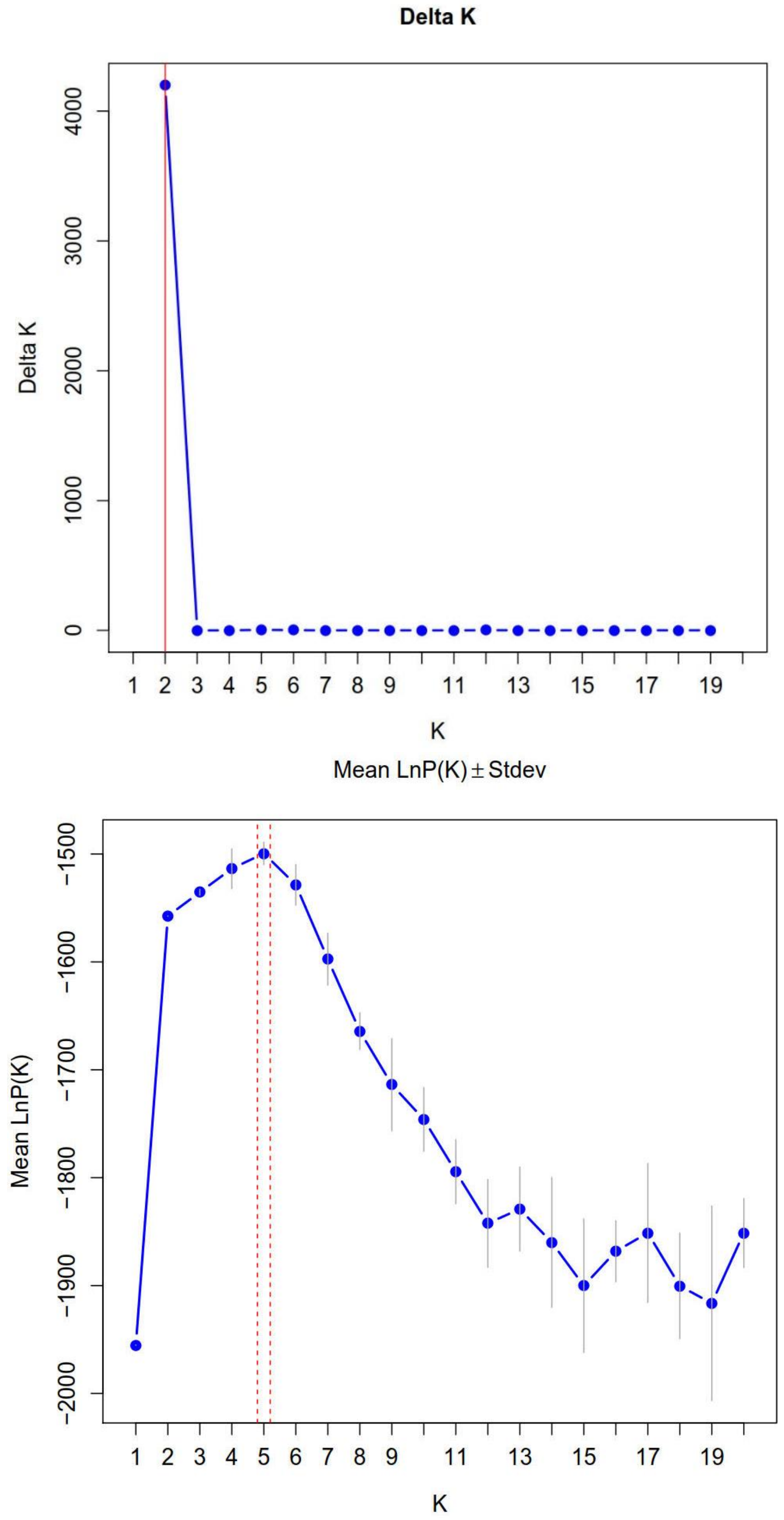

SUPPLEMENTARY FIGURE 6 The estimated number of clusters based on Puechmaille Method (Puechmaille, 2016). The four implanted estimator: 'MedMeaK' (median of means), 'MaxMeaK' (maximum of means), 'MedMedK' (median of medians), 'MaxMedK' (maximum of medians), (subpopulation's arithmetic mean/median membership coefficient threshold set to 0.5). The optimal K (Y-axis) after removing spurious clusters are indicated by red lines, i.e. the number of clusters

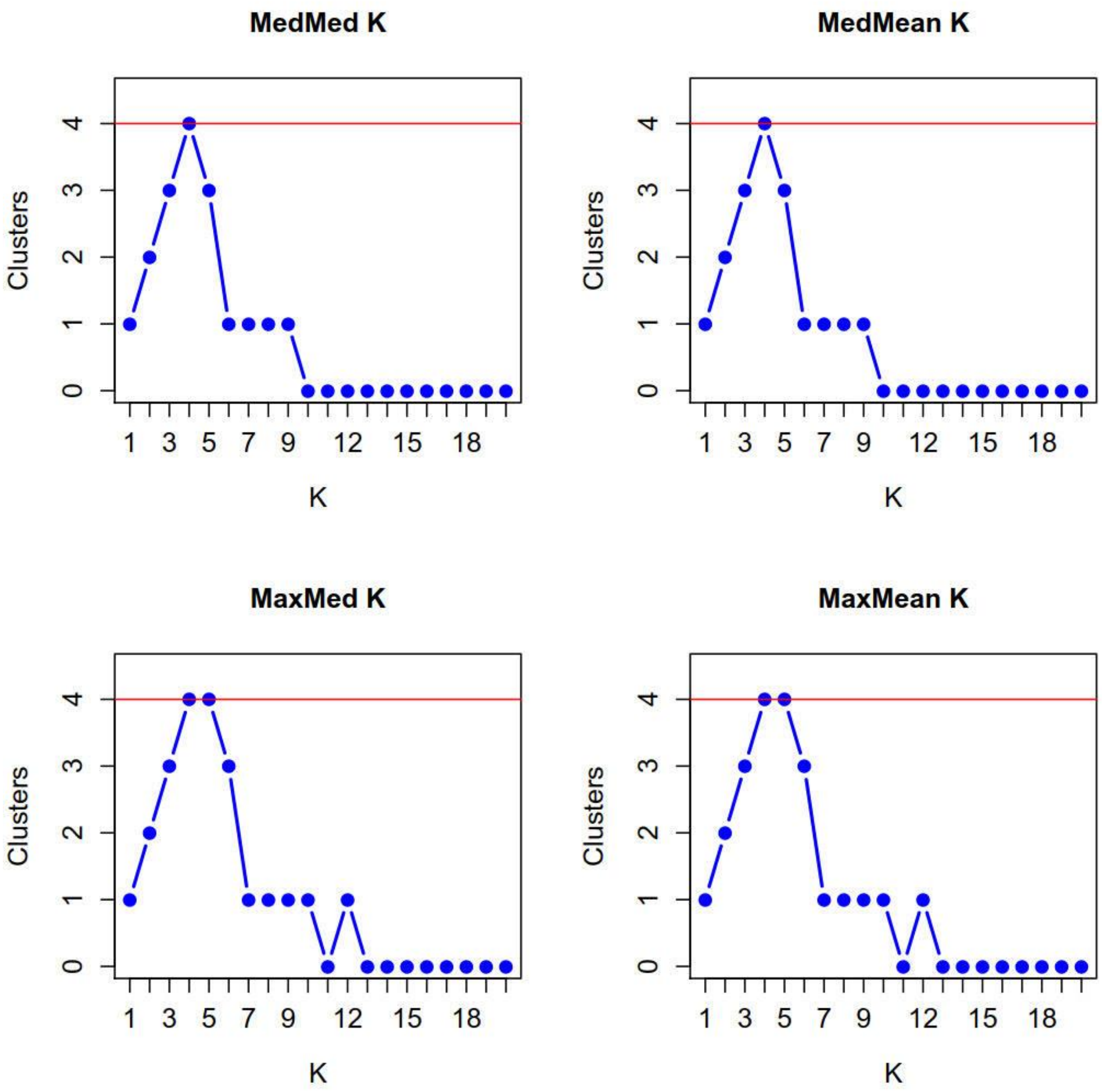

SUPPLEMENTARY FIGURE 7 Statistical measure of goodness of fit as Bayesian Information Criterion (BIC). The optimal clustering solution is indicated by an elbow in the curve of BIC values as a function of K (Jombart & Ahmed, 2011).

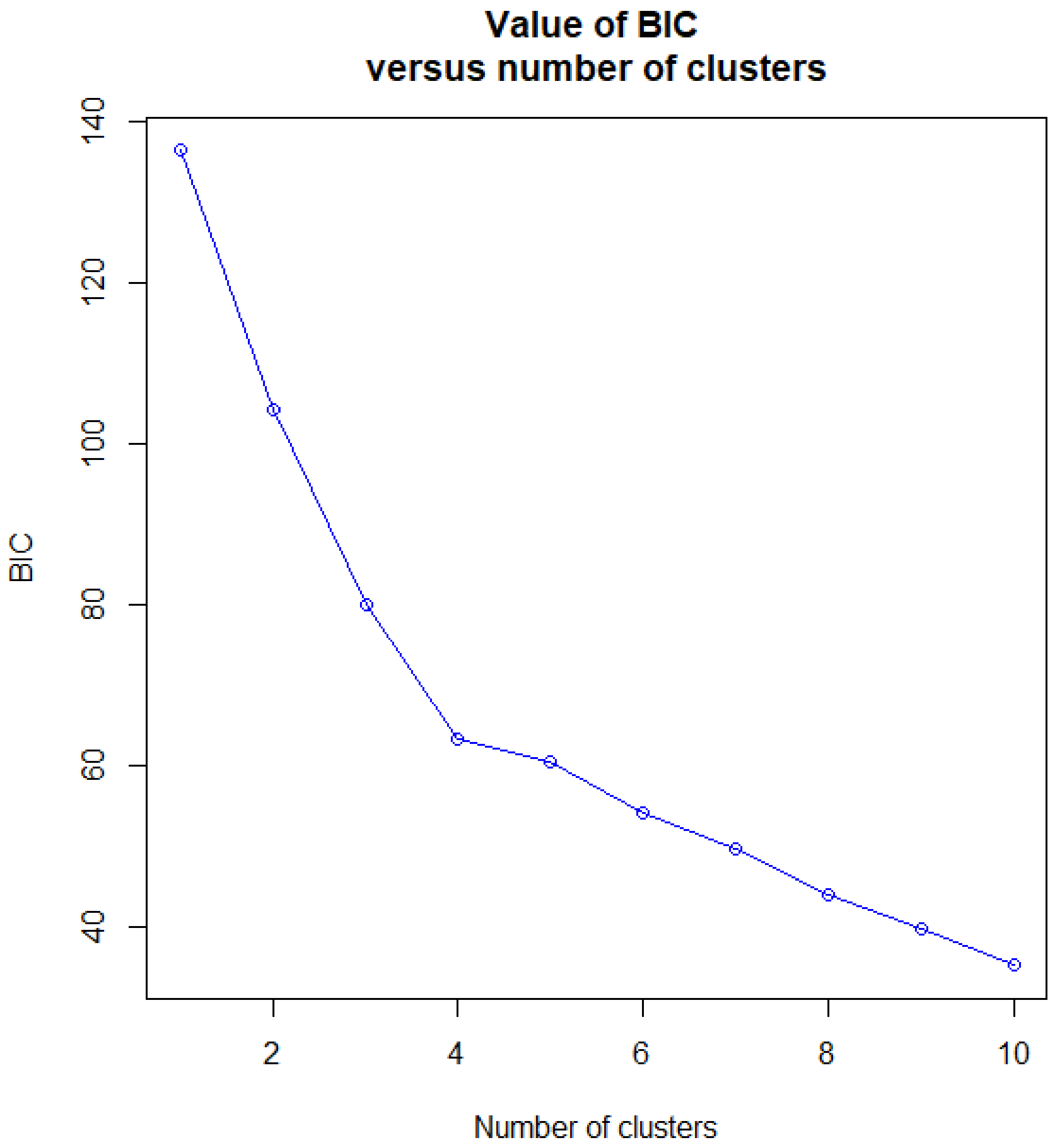

SUPPLEMENTARY TABLE Raw genotype reads of microsatellites generated for this study.

Ind	Pop	Mvis087		Mvi111		Mvis232		G1A		Mer009		Mvis072		Mvis022		Mer005	
Cs3	PanEver	88	90	104	110	146	146	174	174	199	209	277	281	281	285	288	296
Cs5	PanEver	90	90	104	110	152	156	170	170	199	207	265	271	279	281	288	292
Cs10	PanEver	90	90	102	106	146	156	0	0	199	209	265	271	283	283	296	300
Cs11	PanEver	82	82	102	110	146	152	170	174	209	209	271	277	281	281	288	296
Cs15	PanEver	90	90	104	104	146	152	170	174	203	209	271	271	281	281	296	296
Cs16	PanEver	90	94	102	104	146	146	174	174	199	209	271	271	279	285	292	296
Cs17	PanEver	90	96	96	102	152	152	174	174	199	209	271	271	277	281	288	296
Cs18	PanEver	90	90	102	106	146	152	168	174	199	209	271	277	277	279	292	292
Cs19	PanEver	90	96	102	104	146	152	170	170	201	209	265	265	277	281	288	294
Cs21	PanEver	90	90	104	104	146	146	170	170	209	209	271	271	277	281	288	296
Cs22	PanEver	90	90	104	104	146	146	174	174	209	209	265	271	285	285	288	296
Cs23	PanEver	90	94	102	106	146	146	168	170	199	207	281	281	277	277	294	294
Cs24	PanEver	82	82	104	104	152	152	170	174	199	203	265	281	277	283	292	296
Cs25	PanEver	90	90	104	104	146	152	166	174	203	209	271	281	277	285	292	292
Cs26	PanEver	86	92	104	104	146	146	170	170	209	209	265	271	277	283	0	0
Cs28	PanEver	94	94	104	104	146	152	174	174	203	209	263	271	279	285	292	292
Cs29	PanEver	90	90	102	104	146	146	170	174	199	203	265	271	277	283	292	292
Cs31	PanEver	90	94	104	104	146	146	170	170	207	209	263	271	277	279	292	292
26539	PanEver	96	96	104	104	152	152	170	174	199	209	271	277	281	285	288	288
26542	PanEver	90	94	104	110	146	146	174	174	207	209	263	265	279	281	296	296
26548	PanEver	90	90	98	104	146	146	156	156	205	205	265	265	277	285	288	296
23976	PanEver	96	96	98	102	146	146	174	174	199	209	285	287	277	281	288	294
23975	PanEver	96	96	102	102	146	146	174	174	199	209	265	265	281	285	288	294
23974	PanEver	90	96	104	104	146	146	170	174	199	199	265	265	279	281	296	296
24784	PanEver	90	90	102	104	146	146	170	170	0	0	271	273	277	281	296	302
26806	PanEver	90	90	104	104	146	146	170	170	207	209	271	271	277	281	288	296
ZH_130	PanEver	86	90	104	106	146	146	170	170	199	199	269	271	277	281	288	296
ZH_135	PanEver	90	90	102	104	146	146	170	170	209	209	265	265	281	281	288	292
LaE01_2	PanEver	90	96	98	110	146	152	174	174	199	209	281	281	281	281	292	292
26575	PanEver	90	90	104	106	146	152	170	174	205	209	277	281	281	281	296	296
26837	PanEver	90	98	102	104	146	146	162	168	199	209	271	281	281	281	292	296
34843	RusEver	92	96	104	108	146	150	164	170	201	205	269	285	277	281	292	300
35049	RusEver	92	92	98	108	150	154	164	170	205	209	269	269	279	279	292	298
35050	RusEver	92	94	102	104	146	154	170	170	203	207	269	271	275	277	292	298
35093	RusEver	88	94	102	104	150	154	168	168	207	209	267	283	275	281	294	294
DMP01	DanPut	86	86	94	108	156	156	164	164	199	199	271	271	283	283	296	302

DMP02DanPut	80	80	104	110	156	156	160	164	199	199	271	271	281	283	298	298
DMP03DanPut	86	86	106	108	156	156	164	164	199	199	271	273	279	279	300	302
DMP04DanPut	86	86	108	108	156	158	164	164	199	201	267	267	281	283	296	296
DMP05DanPut	86	90	106	106	156	156	166	166	199	199	267	271	279	279	300	300
DMP06DanPut	86	86	102	108	156	156	160	164	199	199	271	273	281	281	298	302
DMP07DanPut	86	86	106	108	156	156	160	162	199	199	267	271	281	281	296	302
DMP08DanPut	86	86	108	108	156	158	156	164	199	199	267	271	283	283	296	300
DMP09DanPut	86	86	108	108	156	158	160	164	199	199	271	271	279	283	294	300
DMP11DanPut	86	86	108	110	156	156	160	162	199	199	267	267	279	283	296	296
DMP12DanPut	86	86	106	108	156	156	160	160	199	199	271	271	275	283	294	300
DMP13DanPut	86	86	102	102	156	156	160	164	199	199	267	271	279	279	300	302
DMP14DanPut	86	86	102	110	156	156	160	160	199	199	271	271	273	279	300	302
DMP15DanPut	86	86	108	110	156	156	160	160	199	199	271	271	279	281	294	298
DMP16DanPut	86	86	94	94	156	156	162	164	199	199	271	271	279	283	300	302
DMP17DanPut	86	86	94	108	156	156	160	164	199	199	271	271	281	281	302	304
DMP18DanPut	86	86	108	108	156	158	160	164	199	199	273	275	279	283	296	304
DMP20DanPut	86	86	94	110	156	156	160	160	199	199	271	271	281	281	300	302
DMP21DanPut	86	88	106	108	156	156	160	160	199	199	271	271	279	283	296	298
DMP22DanPut	86	86	104	108	156	158	160	160	199	199	271	271	279	279	292	302
DMP23DanPut	86	86	106	108	156	158	160	164	199	199	267	271	281	283	298	298
DMP24DanPut	80	80	108	108	156	156	0	0	199	199	271	271	281	283	298	304
DMP25DanPut	86	86	106	108	156	156	160	164	199	199	271	273	281	283	298	302
G2 PanPut	86	86	106	108	156	158	164	164	199	199	271	271	269	283	292	298
26626PanPut	86	96	102	104	146	156	164	170	199	199	271	287	281	281	294	300
26627PanPut	86	86	104	106	156	158	164	164	199	199	269	271	279	285	298	300
P02 PanPut	86	86	106	106	0	0	162	164	199	199	269	271	0	0	292	292
P01 PanPut	86	86	104	112	158	160	164	164	199	199	271	273	277	277	294	294
G1 PanPut	86	86	106	108	156	158	152	164	199	199	267	273	279	283	292	298
Mev3szPanPut	86	86	94	108	156	156	160	164	199	199	271	275	285	285	294	302
MP18 PanPut	86	86	104	104	156	156	0	0	199	199	271	271	277	281	298	298
ZH_117PanPut	86	86	104	106	156	158	164	166	199	199	271	271	283	285	296	298
ZH_128PanPut	86	86	106	106	156	156	160	164	199	199	271	271	281	283	292	300
ZH_129PanPut	86	86	104	106	156	156	162	164	199	199	271	273	279	283	294	294
ZH_138PanPut	86	86	104	106	156	158	164	164	199	199	273	273	275	277	300	302
26835PanPut	86	86	104	104	156	156	164	164	199	199	271	271	277	279	292	296
26735PanPut	86	86	92	106	156	156	162	162	199	199	271	271	281	283	302	302
26581PanPut	86	86	104	106	156	156	164	164	199	199	271	271	285	285	296	298
26714PanPut	86	96	102	104	158	158	162	164	199	199	269	271	281	283	292	294
G9 PanPut	80	80	102	108	156	158	164	164	199	199	271	271	275	285	294	294
